# The pharmacological mechanism of β-elemene in the treatment of esophageal cancer revealed by network pharmacology and experimental verification

**DOI:** 10.1038/s41598-023-38755-w

**Published:** 2023-07-27

**Authors:** Dejiang Zhou, Xiaoling Wu, Xiaoli Liu, Sheng He, Jiang Ni, Beijin Chen, Dong Mu

**Affiliations:** 1 Department of Digestive Medicine, The General Hospital of Western Theater Command, #270, Tianhui Road, Rongdu Avenue, Chengdu, 610000 Sichuan China; 2 Key Laboratory of Molecular Mechanism and Intervention Research for Plateau Diseases of Tibet Autonomous Region, School of Medicine, Xizang Minzu University, Xianyang, 712082 Shaanxi China; 3 Engineering Research Center of Tibetan Medicine Detection Technology, Ministry of Education, School of Medicine, Xizang Minzu University, Xianyang, 712082 Shaanxi China

**Keywords:** Oesophageal cancer, Oncogenesis, Mechanisms of disease

## Abstract

The study aimed to investigate the mechanism of action of β-elemene (ELE) in the treatment of esophageal cancer (EC). In this study, public databases were used to predict related targets in ELE and EC. The network analysis was performed to identify key targets of ELE in EC treatment. Further, bioinformatics and DAVID databases were used for GO and KEGG enrichment analysis, respectively. Ultimately, molecular docking and in vitro cell experiments were conducted to validate the results of network pharmacology enrichment. As a result, 34 candidate targets for ELE in the treatment of EC were obtained, and five key targets (STAT3, EGFR, CTNNB1, BCL2L1 and CASP9) were identified. GO functional annotation yielded 2200 GO entries (*p* < 0.05). KEGG signaling pathway enrichment analysis screened 100 pathways (*p* < 0.05). Molecular docking results showed that ELE had similar affinity with five key targets. In vitro experiments showed that the expressions of STAT3, EGFR and BCL2L1 were significantly decreased, and the expression of CASP9 in the ELE intervention group was significantly increased compared with that in the control group. All in all, ELE may play a key role in the treatment of EC by regulating the expression of STAT3, EGFR, BCL2L1 and CASP9.

## Introduction

Esophageal cancer (EC), one of the most common cancers, has become the leading cause of cancer death^1,2^. According to statistics, the number of new EC cases and deaths worldwide in 2020 were 604,000 and 544,000 respectively, of which 50% of the new cases were in China^3,4^. Studies have shown that race, gene and lifestyle are inextricably linked to the occurrence and development of EC, among which smoking and drinking have become the most important risk factors for increasing the risk of EC^5^. Currently, the main treatment for EC is esophagectomy, but it is highly invasive and may greatly impair patients’ quality of life. In addition, surgical treatment is only suitable for patients with early-stage EC^6^. Unfortunately, most EC patients have already entered the middle and advanced stages of cancer when they seek medical treatment. Compared with 90% for early EC patients after treatment, patients with advanced EC after treatment had an estimated 5-year survival rate of only 6–15%^7^. Thus, the increasing incidence of EC and the poor prognosis after treatment also highlight the importance of continuous in-depth research on the pathogenesis of EC.

Traditional Chinese Medicine (TCM), especially Chinese herbal medicine, has been widely used to treat cancer in China and many other countries. Numerous studies have shown that TCM can not only relieve the symptoms of cancer patients and improve their quality of life, but also reduce the adverse reactions and complications caused by chemotherapy, radiotherapy or targeted therapy^8^. Beta-elemene (ELE) as a sesquiterpene compound extracted from natural Chinese herb *Curcuma wenyujin*^9,10^, which can exert broad-spectrum anti-tumor activity by inhibiting cell proliferation, arresting cell cycle, regulating the expression of apoptotic genes, inhibiting telomerase activity, and inducing apoptosis and autophagy^11,12^. Nowadays, ELE has been widely used in the clinical treatment of various cancers, such as lung cancer^13^, breast cancer^14^, and brain tumors^15^. More recently, a study has also revealed that ELE can inhibit the proliferation and metastasis of EC cells by regulating the phosphorylation of AKT, thereby exerting a therapeutic effect on EC^16^. However, the mechanism and targets of ELE in the treatment of EC still lack further research.

**Figure 1:**
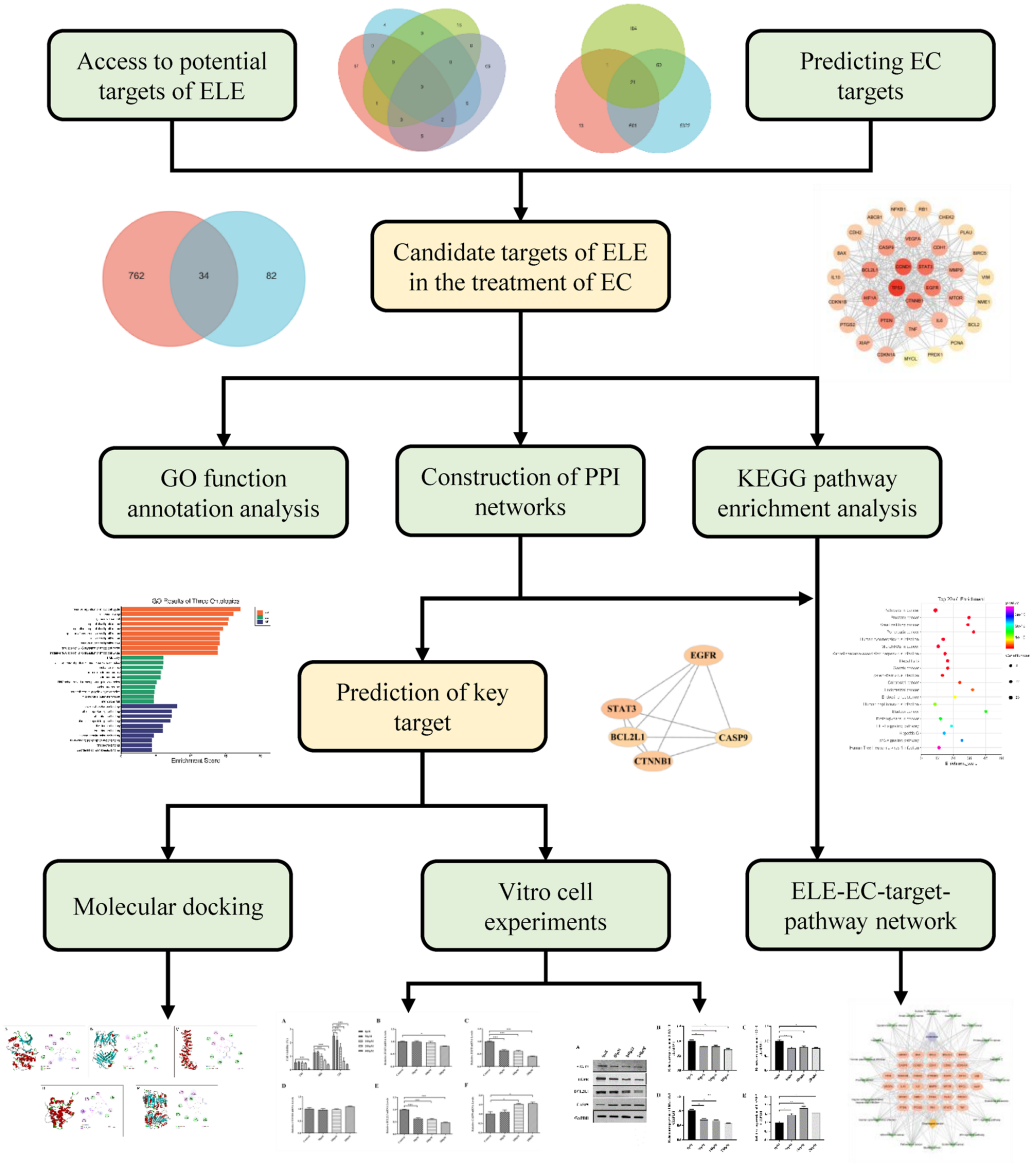
Network pharmacology analysis process.

Network pharmacology is a new research method that breaks the research model of “one drug, one gene, one disease”, and focuses on comprehensively exploring the mechanism of drug treatment of diseases from multiple components, multiple targets, and multiple pathways^17^. Based on this, the study explored the mechanism of action of ELE in the treatment of EC by means of network pharmacology and experimental validation. The related targets of ELE and EC were obtained from massive databases, and key targets were then identified through protein–protein interaction (PPI) network analysis and related literature searches. Finally, the key targets of ELE against EC were verified by in vitro experiments. The network pharmacology analysis process is shown in Fig. 1.

## Results

### Candidate targets of ELE in the treatment of EC

**Figure 2:**
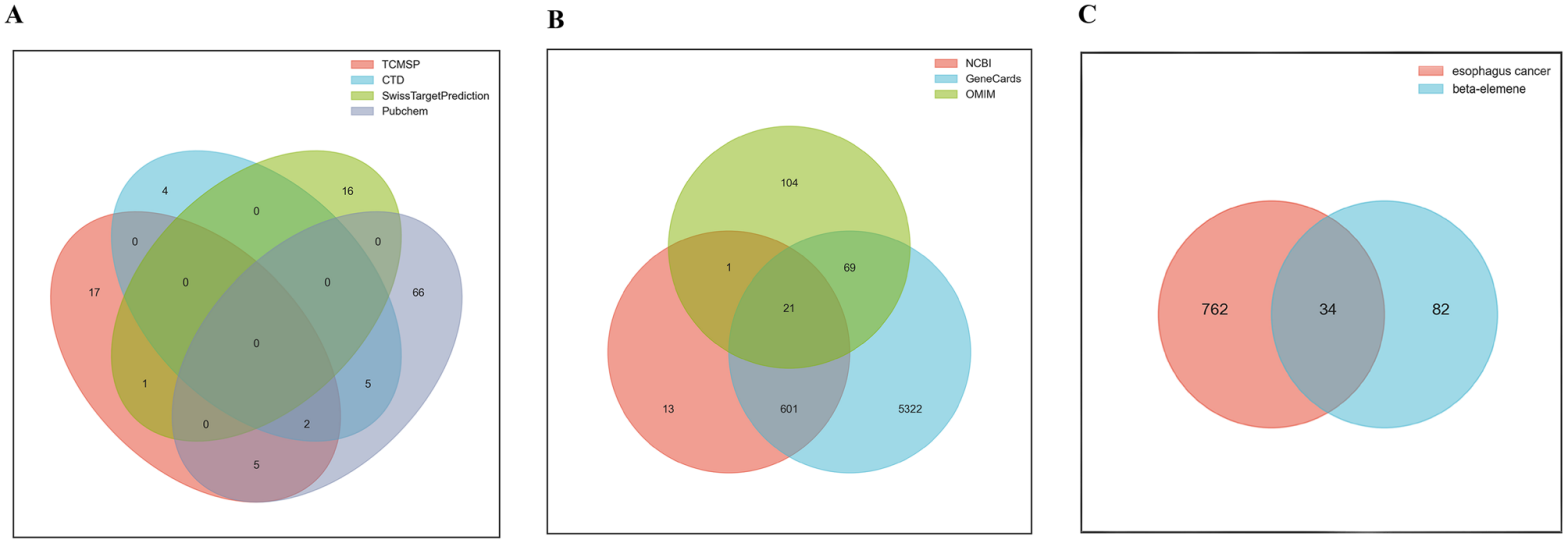
Beta-elemene/esophageal cancer common target acquisition. (**A**) Represents β-elemene target; (**B**) represents esophageal cancer related target; (**C**) represents common target.

The numbers of ELE targets obtained from the TCMSP, CTD, Pubchem and SwissTargetPrediction databases were 25, 11, 17 and 78, respectively. Taking the union of the ELE targets in the above four databases, the final number of ELE targets was 116. The results are presented in Fig. 2A. The EC targets obtained from the NCBI, GeneCards and OMIM databases were 636, 6013 and 195, respectively. The targets of NCBI and GeneCards databases were intersected, and then merged with the targets of OMIM database, and finally, 796 targets of EC were obtained. The results are shown in Fig. 2B. Taking the intersection of the targets of ELE with the targets of EC, 34 candidate targets of ELE for the treatment of EC were finally obtained. The results are exhibited in Fig. 2C.

### PPI network and prediction of key targets

**Figure 3:**
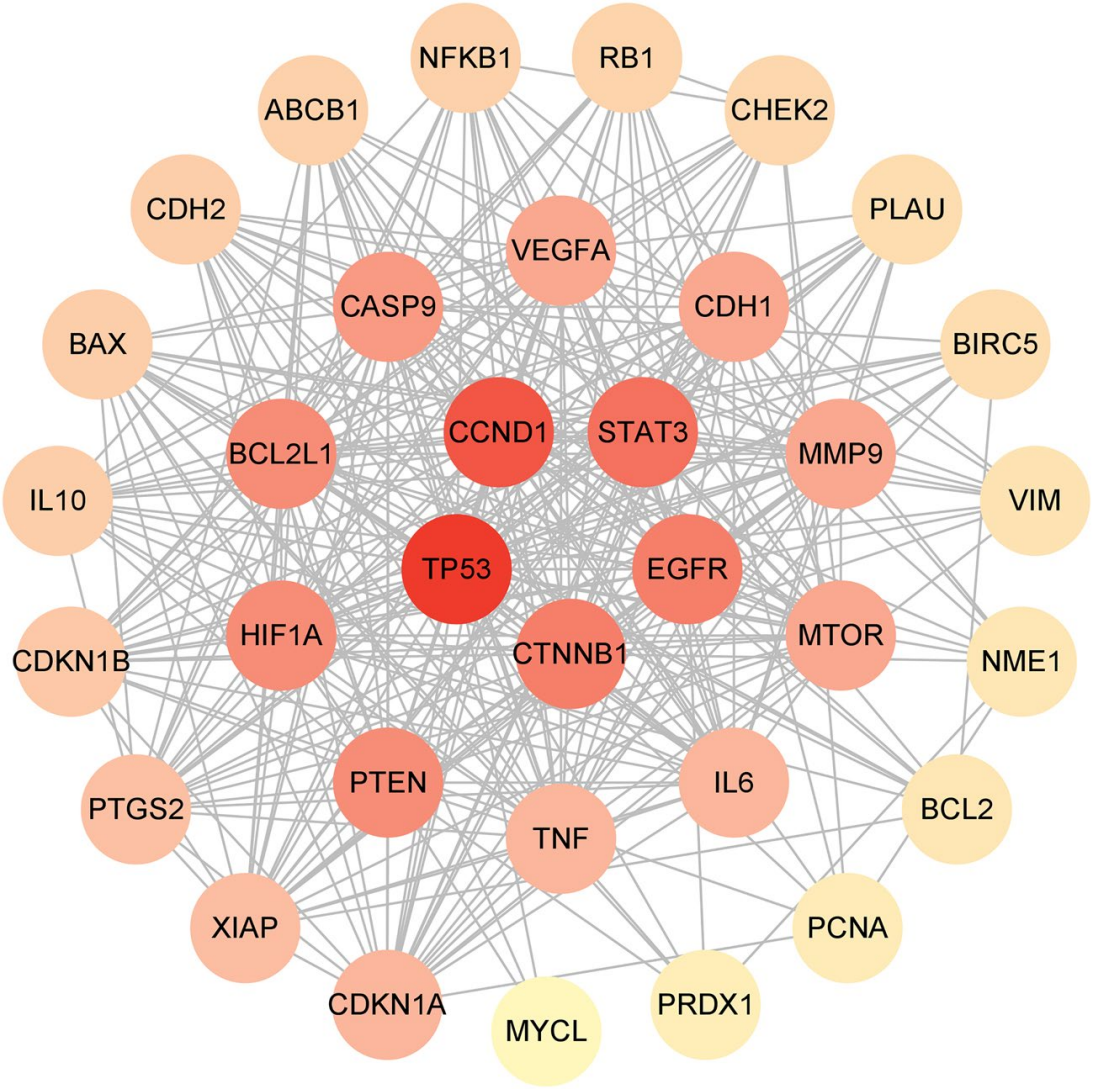
PPI network. The nodes of the target changes from red to yellow, indicating that the degree value decreases in turn.

The PPI network was constructed using the STRING database and visualized by Cytoscape software. As a result, the PPI network contained 34 nodes and 336 edges (Fig. 3). Then, the betweenness centrality (BC), closeness centrality (CC), and degree centrality (DC) of the target were calculated using topological analysis. The top ten center nodes of ELE in the treatment of EC are shown in Table 1. Based on the results of PPI network analysis and literature search, we finally identified STAT3, EGFR, CTNNB1, BCL2L1 and CASP9 as key targets for ELE in the treatment of EC.

**Table 1:** The top ten center nodes of β-elemene in the treatment of esophageal cancer.

Gene name	Betweenness centrality	Closeness centrality	Degree centrality
TP53	0.087	1.000	33
CCND1	0.045	0.943	31
STAT3	0.024	0.892	29
EGFR	0.029	0.868	28
CTNNB1	0.022	0.868	28
BCL2L1	0.016	0.846	27
PTEN	0.037	0.846	27
HIF1A	0.016	0.846	27
CASP9	0.014	0.825	26
CDH1	0.013	0.805	25

### GO and KEGG enrichment analysis

**Figure 4:**
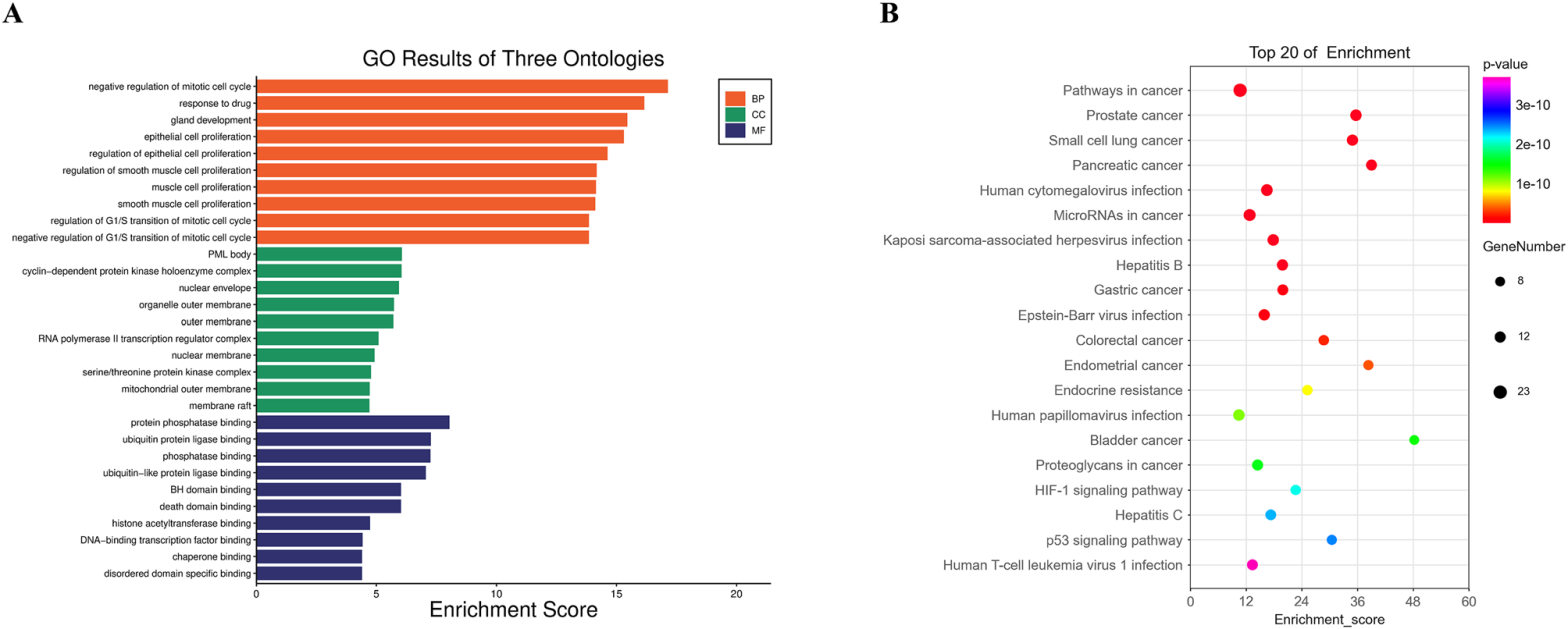
GO functional annotation and KEGG signaling pathway enrichment analysis. (**A**) Represents the GO functional annotation results, including biological process (BP), cellular component (CC), and molecular function (MF), which were represented in red, green, and blue, respectively; (**B**) represents the KEGG signaling pathway enrichment results.

The results of GO functional (biological process (BP), cellular component (CC), and molecular function (MF)) enrichment analysis revealed that 34 candidate targets of ELE for the treatment of EC were enriched in a total of 2200 GO entries (*p* ˂ 0.05), including BP 2010, CC 90 and MF 100. More precisely, BP analysis indicated that candidate targets were mainly concentrated in negative regulation of mitotic cell cycle, response to drug and gland development. CC analysis showed that the candidate targets were mainly concentrated in PML body, cyclin-dependent protein kinase holoenzyme complex and nuclear envelope. MF analysis showed that the candidate targets were mainly concentrated in protein phosphatase binding, ubiquitin protein ligase binding, phosphatase binding. The results of the GO functional annotation are shown in Fig. 4A. In addition, to analyze the enrichment of 34 candidate targets in the corresponding signaling pathways, we performed KEGG signaling pathway analysis, demonstrating that the candidate targets were mainly distributed in 100 signaling pathways (*p* ˂ 0.05), including pathways in cancer, prostate cancer and small cell lung cancer. The results of the KEGG pathway enrichment analysis are shown in Fig. 4B.

### Construction of ELE-EC-target-pathway network

**Figure 5:**
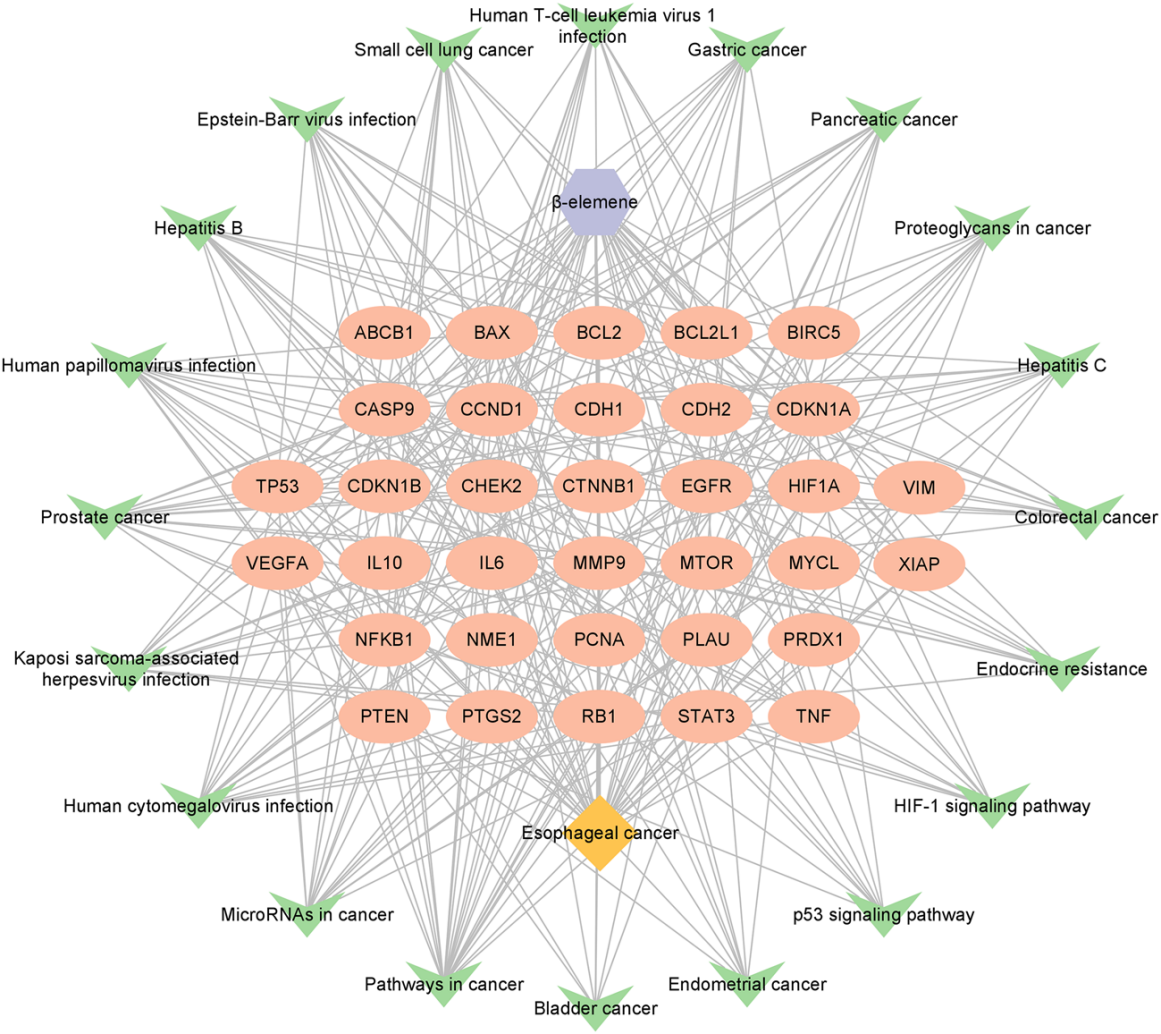
“β-Elemene-esophageal cancer-target-signaling pathway” network construction. Purple hexagonal nodes represent β-elemene; orange diamond nodes represent esophageal cancer; pink circle nodes represent targets; green V-shaped nodes represent signaling pathways.

Taking the 34 candidate targets of ELE in the treatment of EC and the top 20 pathways in the KEGG signal pathway enrichment results as nodes, an ELE-EC-target-signaling pathway network consisting of 56 nodes and 318 edges was constructed using Cytoscape software. Topological network analysis was performed, and the results showed that cancer, microRNAs in cancer and human cytomegalovirus infection signaling pathways have higher degree values. ELE-EC-target-signaling pathway network is shown in Fig. 5.

### Molecular docking of ELE with key targets

**Figure 6:**
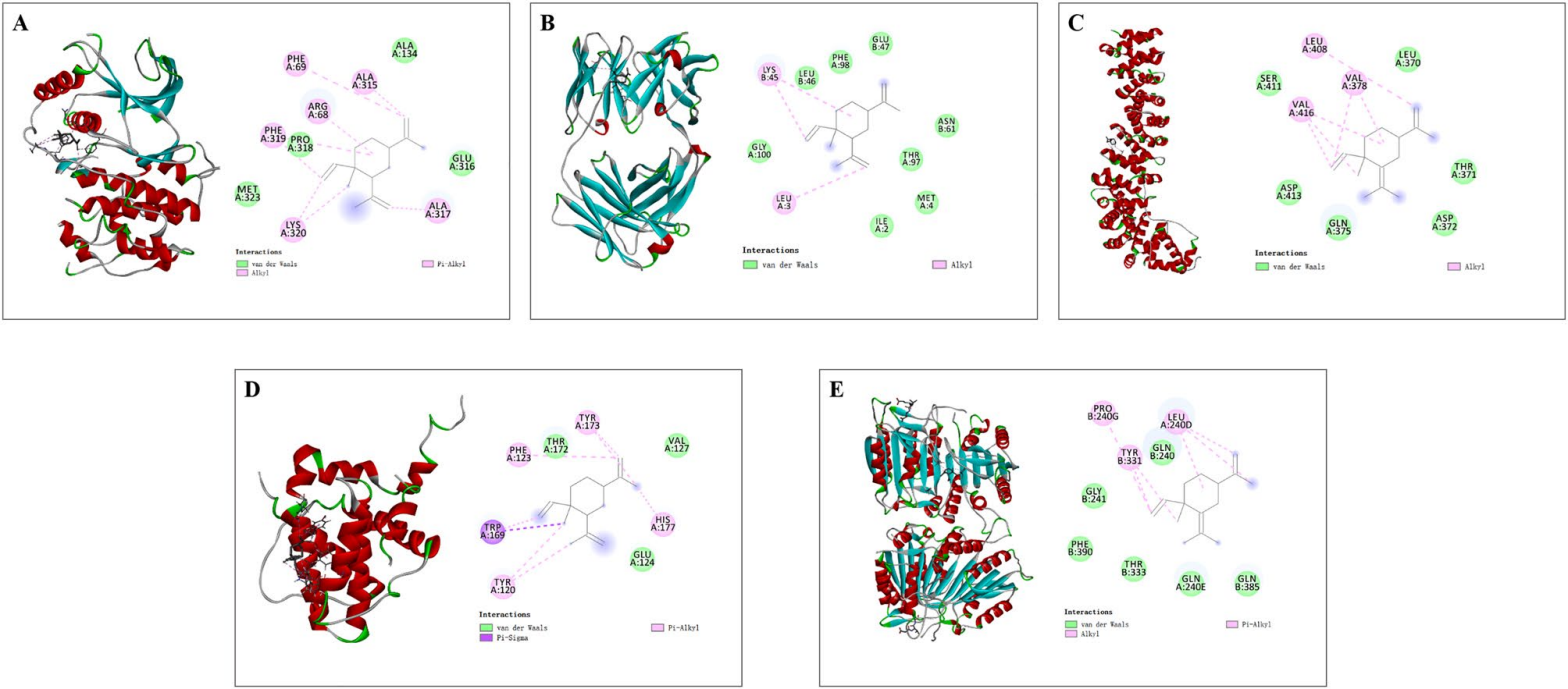
Molecular docking. Molecular models of β-elemene binding to (**A**) STAT3, (**B**) EGFR, (**C**) CTNNB1, (**D**) BCL2L1 and (**E**) CASP9 were shown as 3D and 2D plots.

Molecular docking of ELE with its key candidate targets (STAT3, EGFR, CTNNB1, BCL2L1 and CASP9) for the treatment of EC was applied using the Discovery studio software. As a result, the binding energies for docking with ELE in this study were STAT3 (− 5.2 kJ·mol^−1^), EGFR (− 5.1 kJ·mol^−1^), CTNNB1 (− 5.5 kJ·mol^−1^), BCL2L1 (− 5.3 kJ·mol^−1^) and CASP9 (− 5.4 kJ·mol^−1^). Molecular docking results are shown in Fig. 6.

### The effect of ELE on the viability of ECA-109 cells

**Figure 7:**
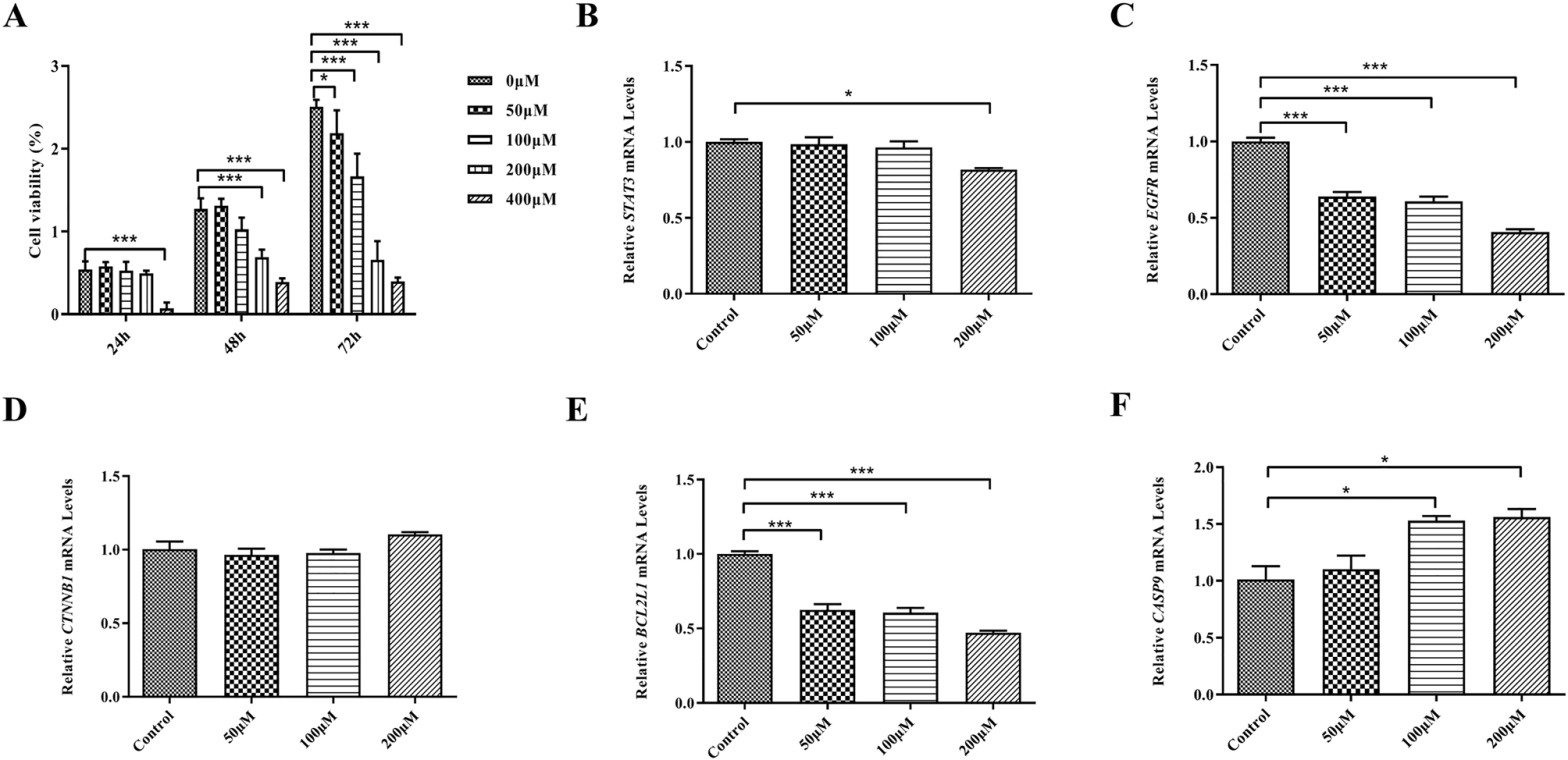
Effects of β-elemene on cell viability and mRNA expression of key targets in ECA-109 cells. (**A**) Represents the results of cell viability experiments; (**B**–**F**) represent the results of the effects of β-elemene on *STAT3*, *EGFR*, *CTNNB1*, *BCL2L1* and *CASP9* mRNA expressions in ECA-109 cells, respectively.

To evaluate the effect of ELE on the viability of ECA-109 cells, cells were treated with ELE at concentrations ranging from 0 to 400 μM for 24–72 h, and the cell viability was then measured using CCK8. The results are shown in Fig. 7A. When ECA-109 cells were treated with 200 μM of ELE, the viability of ECA-109 cells was significantly decreased in a dose-dependent manner. Therefore, 200 μM was used as the maximum dose in subsequent experiments.

### Effects of ELE on mRNA expression levels of key targets in ECA-109 cells

In order to preliminarily study the mechanism of action of ELE in the treatment of EC, we treated ECA-109 cells with different concentrations of ELE for 24 h, and then detected mRNA expression levels of key targets by RT-qPCR. As shown in Fig. 7B–F, compared with the control group, in the ELE intervention group, the mRNA expression levels of *STAT3*, *EGFR* and *BCL2L1* were significantly decreased; the mRNA expression of *CASP9* was significantly increased; and the expression of *CTNNB1* was not significantly different.

### Effects of ELE on protein expression levels of key targets in ECA-109 cells

**Figure 8:**
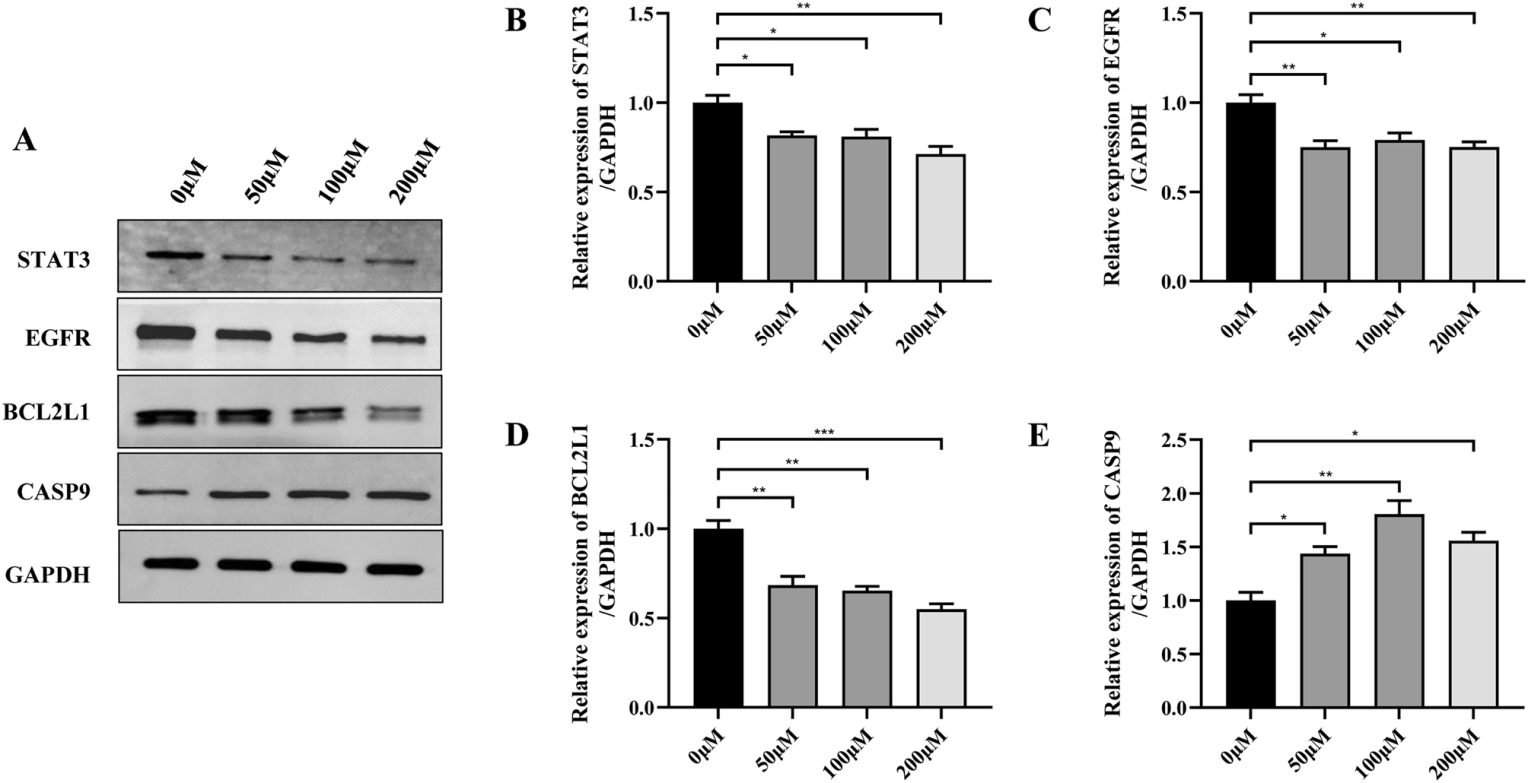
Effects of β-elemene on protein expression levels of key targets in ECA-109 cells. (**A**) Represents the protein expression levels of STAT3, EGFR, BCL2L1, and CASP9 analyzed by western blot. (**B**–**E**) Represents the statistical bar graph of quantification analysis of protein bands in (**A**).

To further clarify the effect of ELE on the expression of key targets in ECA-109 cells, STAT3, EGFR, BCL2L1 and CASP9, which were statistically significant with regard to the mRNA expression levels between the two groups, were selected for further study, and western blot was used to detect the protein expression levels of STAT3, EGFR, BCL2L1 and CASP9 after ELE intervention. As shown in Fig. 8, the results showed that the protein expression levels of STAT3, EGFR and BCL2L1 in the ELE intervention group were significantly reduced, while the protein expression levels of CASP9 were significantly increased compared with the control group.

## Discussion

This study investigated the mechanism of action of ELE in EC treatment via network pharmacology analysis and experimental validation. As a result, we identified five key candidate targets for ELE in the treatment of EC, namely STAT3, EGFR, CTNNB1, BCL2L1 and CASP9. Then, in vitro cell experiments confirmed that ELE may play a certain role in the treatment of EC by down-regulating the expression levels of STAT3, EGFR and BCL2L1 and up-regulating CASP9 expression.

The theory of TCM holds that human body is an organic whole, and the treatment of diseases needs to be comprehensively considered from multiple angles and aspects, in order to better play the role of drugs in symptomatic treatment and delaying the development of diseases. Network pharmacology is a brand-new discipline that integrates systems biology and bioinformatics. It focuses on systematically excavating the mechanism of drug treatment of diseases as a whole, which is consistent with the concept of TCM for disease treatment^18^. Ancient physicians believed that the etiology and pathogenesis of EC were deficiency, blood stasis, heat, and toxin, and the treatment should be used to invigorate Qi and promote blood circulation, strengthen spleen and kidney, regulate Qi and resolve phlegm, clear heat and detoxify^19^. The TCM *Curcuma wenyujin* has the effects of promoting Qi and relieving depression, promoting blood circulation and relieving pain, and promoting gallbladder and removing jaundice, and is widely used in the clinical treatment of lung cancer, liver cancer, breast cancer, pancreatic cancer, EC, etc. Beta-elemene, as the active compound extracted from *Curcuma wenyujin*, plays a key role in the treatment of various cancers including esophageal cancer, but the mechanism of action remains to be further studied^20^. Therefore, this study explored the mechanism of action of ELE in the treatment of EC based on network pharmacology, which may provide a new method for the treatment of EC.

To explore the key targets of ELE in the treatment of EC, this study integrating PPI network analysis with literature search revealed that STAT3, EGFR, CTNNB1, BCL2L1 and CASP9 may be core targets. Among them, the core target with a higher degree value was STAT3. Research has shown that STAT3 is a very important transcription factor of the STAT family, which is thought to be involved in the regulation of various key functions, including cell survival, differentiation, apoptosis and metastasis, and is overexpressed in multiple cancer cells^21^. Besides, studies have also found that inhibiting the expression of STAT3 can induce apoptosis and G1 cell cycle arrest in EC ECA109 cells, and inhibit cell migration^22^. Our result also found that ELE intervention was able to significantly reduce STAT3 expression in ECA-109 cells, which further suggested that STAT3 may be a key target of ELE for the treatment of EC.

Besides, EGFR, BCL2L1 and CASP9 also have high degree values in the PPI network. EGFR, a tyrosine kinase receptor in the ErbB family, is involved in various processes of cancer progression, including cell proliferation, migration and metastasis^23,24^. It has been reported that EGFR is highly expressed in a variety of cancers, such as lung cancer, breast cancer, colorectal cancer^25^. It is worth noting that the increased expression of EGFR in EC is closely related to cancer metastasis and poor prognosis^26^. BCL2L1, also known as BCL-xl, is one of the BCL-X subtypes belonging to the BCl2 family, and the other subtype is BCL-xs. Interestingly, the two have opposite effects, with BCL-xl being anti-apoptotic and BCL-xs being pro-apoptotic^27^. Currently, BCL2L1 has been found to be highly expressed in a large number of cancers, including myeloma, lymphoma, liver cancer, gastric cancer, and ovarian cancer^28^. CASP9, a cysteine-aspartic protease, plays a crucial role in regulating cell differentiation, proliferation, and apoptosis, and is associated with the onset and progression of various cancers^29^. One study has showed that Tanshinone IIA could significantly induce apoptosis and inhibit the proliferation of human EC Eca-109 cells in vitro by up-regulating the expression of CASP9^30^. Taken together, these data further support our view of the importance of ELE in the treatment of EC.

In conclusion, through network pharmacology and validation experiments, we have revealed that ELE may play a role in the treatment of EC by regulating the expression of STAT3, EGFR, BCL2L1, and CASP9. It should be noted that this study also has some limitations, namely, this study uses a data mining approach to explore the possible mechanisms of ELE in the treatment of EC, but further in vivo animal experiments are needed to confirm the findings.

## Methods

### Access to potential targets of ELE

Potential targets of ELE were obtained from the TCMSP (https://old.tcmsp-e.com/tcmsp.php), CTD (http://ctdbase.org/), Pubchem (https://pubchem.ncbi.nlm.nih.gov/) and SwissTargetPrediction (http://swisstargetprediction.ch/) databases. Among them, targets were obtained from the SwissTargetPrediction database through the following two processes: First, the Canonical SMILES structure of ELE was copied from Pubchem. Second, the SMILES structure was input into the SwissTargetPrediction search box to predict the targets of ELE.

### Predicting EC targets

Targets related to EC were obtained from the NCBI (https://www.ncbi.nlm.nih.gov/), GeneCards (https://www.genecards.org/) and OMIM (https://www.omim.org/) databases.

### Construction of PPI networks

The candidate target list of ELE in the treatment of EC was input to the STRING database (https://string-db.org/), and the species was selected as “Homo sapiens” to construct a PPI network. The “string_interactions file” was downloaded from the PPI network analysis results of the STRING database, and the visualization and topology analysis of the PPI network were then performed using Cytoscape 3.9.1 software.

### GO functional annotation and KEGG pathway enrichment analysis

The candidate target list of ELE in the treatment of EC was uploaded to the bioinformatics platform (http://www.bioinformatics.com.cn/), and Gene Ontology (GO) functional annotation was performed using the GO enrichment analysis function of the bioinformatics platform. Similarly, the candidate target list of ELE for the treatment of EC was uploaded to the file selection column of the DAVID database (https://david.ncifcrf.gov/) for Kyoto Encyclopedia of Genes and Genomes (KEGG) pathway enrichment analysis.

### Construction of ELE-EC-target-pathway network

The one-to-one correspondence of ELE-EC-target-signaling pathway was sorted out in EXCEL, and the network of ELE-EC-target-signaling pathway was constructed using Cytoscape 3.9.1. Among them, the top 20 signaling pathways ranked by *p* value were incorporated into the ELE-EC-target-signaling pathway network.

### Molecular docking

Molecular docking of receptor protein with ELE was performed using the Discovery studio software. The 3D structures of all receptor proteins were obtained from the RCSB PDB (https://www.rcsb.org/) database, and the 3D chemical structures of ELE were obtained from the PubChem database.

### Cell culture

The human EC cell line ECA-109 was purchased from Shanghai Cyberkang Biotechnology Co., Ltd. The ECA-109 cells were added to RPMI-1640 (cat. no. PM150110; Procell Life Science and Technology Co., Ltd) supplemented with 10% fetal bovine serum and 1% antibiotics (100 U·mL^−1^ penicillin and 100 U·mL^−1^ streptomycin), and cultured in an incubator containing 5% CO_2_ at 37 °C. After the cells entered the rapid growth phase, subculture and follow-up experiments were performed.

### Cell viability assay

Cell viability was determined by Cell Counting Kit (CCK-8) assay. Briefly, 2000 ECA-109 cells/well were seeded in a 96-well plate, and after cells were fully attached, cells were then treated with ELE (cat. no. HY-107324; MedChemExpress LLC, Shanghai, China) at different concentrations (0 μM, 50 μM, 100 μM, 200 μM, and 400 μM) for 24, 48 and 72 h. Afterwards, discard the original medium in the 96-well plate, add 10 µL of pre-prepared 10% CCK-8 solution (cat. no. HY-K0301; MedChemExpress LLC, Shanghai, China), and incubate in a 37 °C incubator for 0.5–4 h until the color turns orange. Finally, the absorbance value of each well at 450 nm wavelength was measured with a microplate reader.

### RNA extraction and RT-qPCR analysis

TRIzol (cat. no. T9424; Sigma-Aldrich LLC, Shanghai, China) was used to extract total RNA from cell samples. Nano Drop 2000 (Thermo Scientific, Inc.) was used to detect RNA concentration and purity. Reverse transcription kit (Takara Biomedical Technology (Beijing) Co., Ltd) was used for reverse transcription. And ABI 7500 instrument (Applied Biosystems, Shanghai, China) was employed for fluorescence quantitative detection. Using GAPDH as a reference, the relative mRNA expression levels of *STAT3*, *EGFR*, *CTNNB1*, *BCL2L1* and *CASP9* were analyzed. The primer list and sequence are presented in Table 2.

**Table 2:** List of primers and sequences.

Number	Gene	Sequences
1	STAT3-F	CTTTGAGACCGAGGTGTATCACC
2	STAT3-R	GGTCAGCATGTTGTACCACAGG
3	EGFR-F	CCCTCCTGAGCTCTCTGAGT
4	EGFR-R	ATGCTGTCCTCAGTCAAGGC
5	CTNNB1-F	CACAAGCAGAGTGCTGAAGGTG
6	CTNNB1-R	GATTCCTGAGAGTCCAAAGACAG
7	BCL2L1-F	GCCACTTACCTGAATGACCACC
8	BCL2L1-R	AACCAGCGGTTGAAGCGTTCCT
9	CASP9-F	GTTTGAGGACCTTCGACCAGCT
10	CASP9-R	CAACGTACCAGGAGCCACTCTT
11	GAPDH-F	GGAGCGAGATCCCTCCAAAAT
12	GAPDH-R	GGCTGTTGTCATACTTCTCATGG

### Western blot analysis

The protein expression levels of STAT3, EGFR, BCL2L1 and CASP9 in ECA-109 cells were measured by western blot analysis. Specifically, ELE-treated ECA-109 cells were collected and total cell proteins were extracted in RIPA buffer (Beyotime, Shanghai, China) for 30 min on ice. Protein concentrations were measured using a BCA protein assay kit (cat. no. P0012; Beyotime, Shanghai, China) according to the manufacturer’s instructions. The proteins were resolved by SDS loading buffer and denatured at 95 °C for 5 min. Subsequently, a total of 30 μg of proteins were separated by SDS-PAGE on 10% gels, which were then transferred onto PVDF membranes and blocked with 5% skim milk for 2 h at room temperature. The PVDF membranes were incubated with the corresponding primary antibodies (STAT3, EGFR, BCL2L1 and CASP9 (ZEN BIO, China); GAPDH (Proteintech, USA)) overnight at 4 °C. After washing with TBST, the PVDF membranes were incubated with secondary antibody overnight at room temperature. Finally, detection was routinely performed with a chemiluminescent HRP substrate (Beyotime, Shanghai, China) and an ECL imaging system (Tanon, Shanghai, China). The result of gels images was cropped and full-length gels and blots are included in the Supplementary Data.

### Statistical analysis

SPSS 22.0 was used for statistical analysis of experimental data. Data were presented as mean ± standard deviation (SD), and differences between multiple groups were assessed using one-way analysis of variance (ANOVA). A *p*-value < 0.05 was considered statistically significant.

## Conclusion

In summary, the present study is the first to systematically explore the possible mechanisms of ELE in the treatment of EC using network pharmacology approach and in vitro validation experiments. The network pharmacological analysis predicted that ELE may exert its therapeutic effects against EC via regulating multiple targets and pathways. In vitro validation experiments confirmed that the possible mechanism of ELE treating EC was to down-regulating the expression of STAT3, EGFR and BCL2L1, and up-regulating the expression of CASP9, thereby improving the progression of EC. This finding may serve as a reference to study the mechanism of action of ELE in the treatment of EC and to supply novel targets for the treatment of EC.

## Supplementary Information

Supplementary Figure 1.

## Data Availability

The data that support the findings of this study are available from the corresponding author upon reasonable request.
