# Supplementary material for: The pharmacological mechanism of β-elemene in the treatment of esophageal cancer revealed by network pharmacology and experimental verification
Source: Sci Rep. 2023 Jul 27;13:12160. doi: 10.1038/s41598-023-38755-w (PMC10374640; doi:10.1038/s41598-023-38755-w)

Supplementary Information

In this study, the images of all blots have been provided as they were, with visible membrane edges, and a guarantee can be made that the results are real and valid.

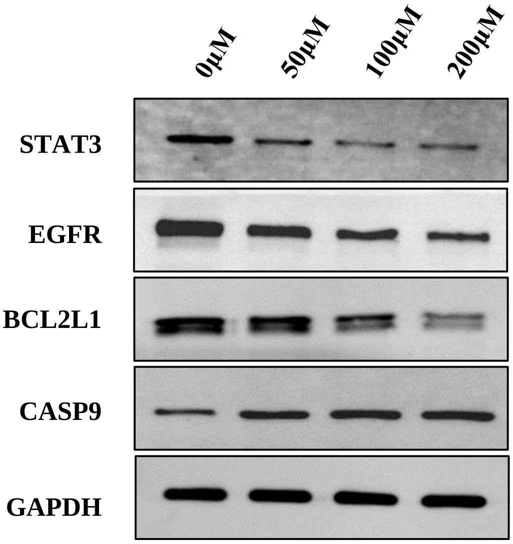

Figure 8A

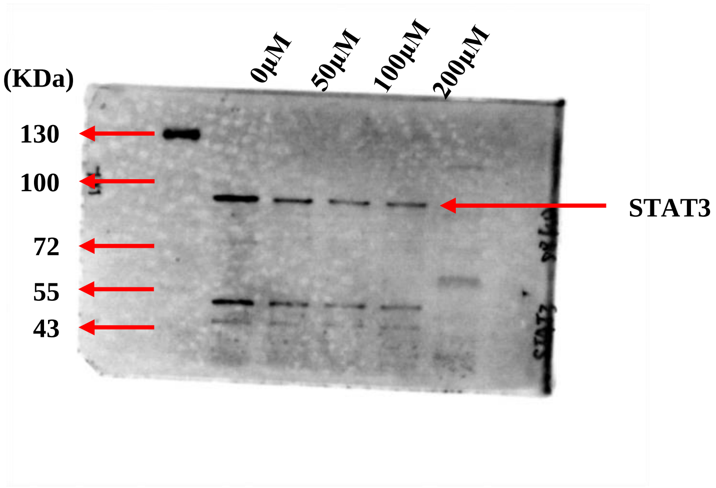

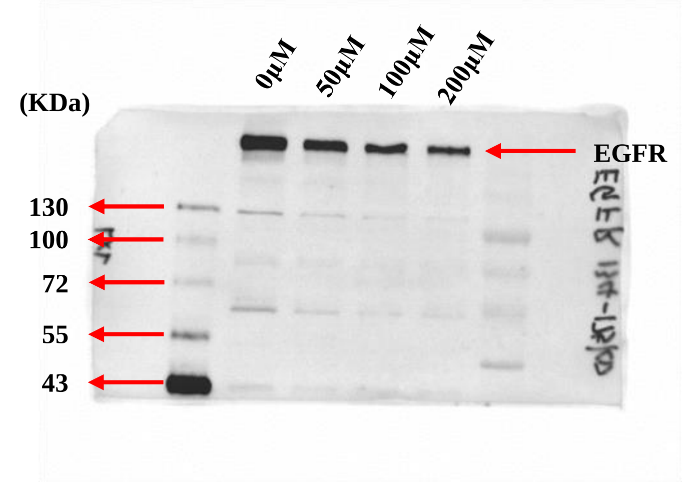

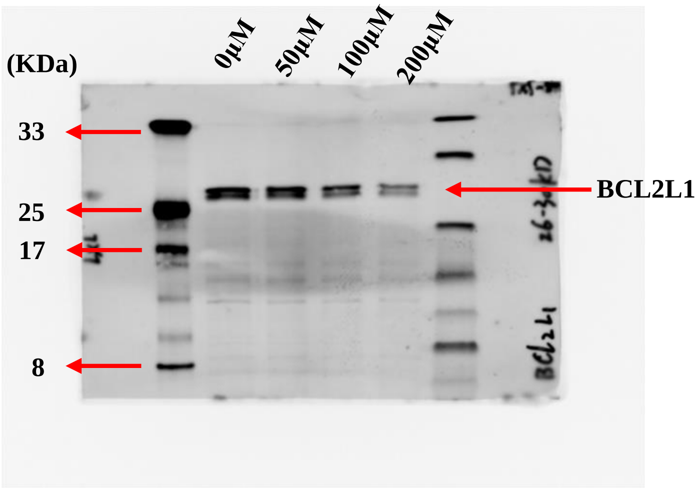

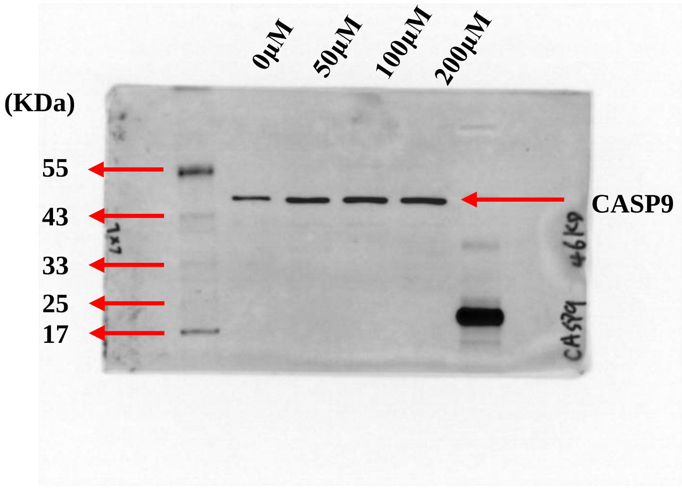

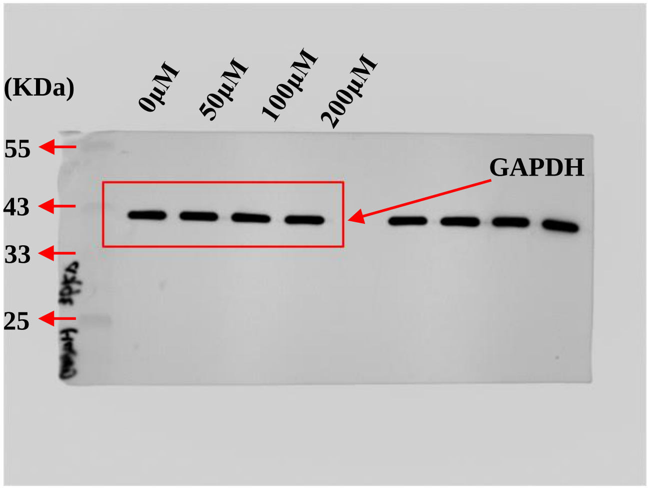

Supplement: Supplementary file 1 — Supplementary Figure 1. [file 41598_2023_38755_MOESM1_ESM.pdf]
